# Depression Literacy and Self-Reported Help-Giving Behaviour in Adolescents in Ireland

**DOI:** 10.1007/s10578-024-01727-w

**Published:** 2024-06-25

**Authors:** Sadhbh J. Byrne, Lorraine Swords, Elizabeth Nixon

**Affiliations:** 1 Department of Psychology, Maynooth University, Maynooth, Ireland; 2 School of Psychology, Trinity College Dublin, Dublin, Ireland

**Keywords:** Adolescents, Mental health, Depression, Mental health literacy, Depression literacy

## Abstract

This questionnaire-based study aimed to explore depression literacy (DL) and help-giving experiences in 12–18-year-old adolescents (*N* = 535, *M*_age_ = 14.9 years, 51.8% male) in Ireland. In response to a vignette depicting a character displaying symptoms of depression, 46.7% labelled these symptoms ‘depression’, with increasing age and empathy associated with increased likelihood of labelling as such. Almost all (92.1%) believed the character needed help, but the perceived helpfulness of potential responses varied. Over one-third (38.2%) indicated they knew someone who had displayed similar symptoms in the past year, with 85.2% of these reporting having helped this person. Reported help-giving responses included comforting the person, encouraging professional help-seeking, and informing an adult. The findings suggest adolescent DL can be best supported by developmentally-sensitive interventions that encourage empathy and the importance of engaging adults’ assistance.

**Supplementary Information:**

The online version contains supplementary material available at 10.1007/s10578-024-01727-w.

Adolescence is regarded as a window of vulnerability for the development of psychopathology, with the first onset of many mental disorders occurring by the end of this developmental period [1, 2]. Experiencing mental health difficulties at a young age can foreshadow recurrence and significant related impairments throughout the lifespan [e.g., 3]. Depression is among the most common mental disorders experienced by adolescents, and reports suggest it is becoming more prevalent among young people in recent years [4]. However, some suggest that depression is under-recognised and, consequently, often goes untreated professionally [5].

Peers are an important potential source of support for adolescents [e.g., 6]; studies have demonstrated that if adolescents do seek help for their mental health, they first turn to family and friends [e.g., 7]. For example, Doyle et al. [8] reported that the most commonly-named person that participants felt they could talk to about issues that bothered them was a friend (83%). It is therefore important to understand the nature of the help that may be provided by peers if sought. Moreover, it has been suggested that members of a young person’s social network may be able to detect signs of distress, and reach out and provide help, *before* it is sought by the young person themselves [9]. While the provision of mental health support to adolescents “is ideally provided by adults, who have a greater capacity to solve problems, take on caring responsibilities and provide practical support” [10], provision of informal peer support regularly occurs in adolescence; in Mason et al.’s [11] study, 97.5% of adolescents reported that they offered help when they had contact with an individual experiencing mental health difficulties in the previous 3 months.

Although the literature is clear that peers are perceived as a principal source of support for adolescents in distress, knowledge of what adolescents actually *do* to support peers is comparatively scant [12–14]. Studies have primarily focused on university students [e.g., 15] and on young people’s responses to a peer’s disclosure of suicidal ideation [16], while research with younger adolescents, or where depression occurs without suicidal ideation, is lacking. Furthermore, many studies report on hypothetical help-giving or help-giving intentions [e.g., 17], with little research on the nature of help provided in real-life situations. It is therefore necessary to further explore the support that adolescents report providing to others experiencing mental health challenges, including depression. As such, the current study aims to explore adolescents’ self-reported exposure to individuals showing signs of depression, whether they report having provided help, and, if so, the nature of this help.

A potential contributing factor to whether adolescents engage in help-giving is their level of mental health literacy [MHL; e.g., 18]. MHL was originally defined as the “knowledge and beliefs about mental disorders which aid their recognition, management or prevention” [19] and as such may potentially influence whether an adolescent deems a peer in need of help. This definition has expanded in the years since, such that conceptualisations of MHL and how it is measured has been found to vary across studies [20, 21]. In an updated definition of MHL, help-giving itself is addressed, through the inclusion of “first aid skills”; “mental health first aid” being defined in this context as “the help provided to a person developing a mental health problem or in a mental health crisis” [22, 23]. Researchers have also suggested that applying the concept of MHL to specific disorders may help to focus efforts for intervention [24, 25]; for example, assessing depression literacy (DL) specifically may have more practical use than an assessment of MHL more broadly conceived. Efforts to improve DL during adolescence have the potential to increase the likelihood that young people will recognise their own problem, or a peer’s problem, and seek out appropriate help [18]. Thus, assessing existing levels of knowledge among adolescents is an important first step. The present study therefore seeks to extend research on MHL by focusing specifically on DL among adolescents, as well as exploring help-giving behaviour.

There are indications that adolescents’ perceptions of their own MHL tend to be very positive. For example, in one Scottish study, the overwhelming majority of adolescents (92% of male adolescents and 96% of female adolescents) stated that they felt they had a good understanding of depression [26]. However, research also indicates that adolescents’ accounts of their knowledge of mental health and mental illness demonstrate both confusion and underlying stigma; for example, in one study adolescent participants expressed concerns about the ‘authenticity’ of others’ mental health issues, due to a perceived ambiguity of symptoms and lack of visible ‘proof’ [27].

While the lack of clarity around the conceptualization of MHL makes comparison difficult [21], a frequently-used component of MHL assessments involves examining how participants label symptoms presented in a vignette. Recognition of the symptoms that are in line with the described disorder, and labelling the disorder, is interpreted as indicative of higher MHL. While some researchers have asserted that centring the conceptualisation of MHL on recognition of psychiatric labels may have negative consequences (see [21] for a discussion), labelling has been found to predict less stigmatising attitudes towards individuals with mental illness [28], and the model of youth help-seeking developed by Srebnik et al. [29] posits problem recognition as the first step. Previous research has also found associations between adolescents’ labelling symptoms as ‘depression’ and increased likelihood of recommending professional help [30–32]. It therefore appears that assessing adolescents’ recognition and labelling of symptoms may have some utility.

The available research suggests that rates of recognition and labelling of depression symptoms are low amongst adolescents [33]. A systematic review and narrative meta-synthesis by Georgakakou-Koutsonikou and Williams [34] found identification of depression among young people varied from 23.4 to 73%, with an average of 40.8%. These figures may be lower among non-Western populations; for example, one study [35] found that only 5% of Nigerian adolescents in their study labelled a vignette character’s symptoms as depression. It appears that information on symptom severity might bear influence. Burns and Rapee [36], for example, found that while two thirds of 15–17-year-olds labelled a vignette character’s symptoms as ‘depression’ when they were also described as having suicidal thoughts, only one third did so when this more extreme symptom was absent. More recently, however, Byrne et al. [37] found that half of adolescent participants did not label a vignette character as ‘depressed’, even when explicit reference to the character’s suicidal ideation was made. Notwithstanding this, a majority of participants in the same study indicated that they believed the depressed character would benefit from the help of a mental health professional, suggesting that they can recognise that certain symptoms are concerning and require assistance, regardless of symptom labels.

The present study therefore aims to extend the literature by investigating adolescents’ DL, through exploring whether they label a cluster of ‘symptoms’ (indicative of depression, as conceptualised in the DSM-IV) as ‘depression’, their recognition of symptoms, assessment of prognosis (length of time until recovery), and beliefs about the helpfulness or harmfulness of potential responses.

In order to better understand adolescents’ help-giving behaviour in this context, it may be useful to explore empathy, as this construct is demonstrably associated with prosocial behaviour more generally [38]. Empathy has traditionally been conceptualized to encompass both ‘state’ or situational affective experiences (such as sympathy and anger) in response to specific scenarios, as well ‘trait’ cognitive abilities to comprehend others’ emotions more generally [39, 40]. Empathy’s role in influencing helping behaviour has been linked with attributions of low perceived controllability, low anger, and high sympathy [41]. In one Irish study with 14- to 17-year-old adolescents, high sympathy, low anger, and low perceived controllability of symptoms predicted greater acceptance of a hypothetical peer with depression [42], however ‘trait’ empathy and help-giving responses were not assessed. Indeed, although empathy has long been linked broadly to prosocial and helping behaviour, to the best of the authors’ knowledge, no study to date has specifically investigated the relationship between adolescents’ empathy and help-giving in the context of providing support to an individual with depression.

The links between empathy and MHL are also not yet well-understood, with very limited extant research. One study has demonstrated that adults with greater MHL have greater trait empathy [43]. A study of U.S. college students found that participants with higher empathy rated both emotional and behavioural signs of suicide as being more serious [44]. Empathy has been explored in relation to adolescent stigma; for example, a study with Irish adolescents found significant associations between higher levels of trait empathy and lower stigma-related responses towards vignette characters with depression [45]. Given the extremely limited literature currently available, the relationship between both state and trait empathy and DL in adolescents requires further exploration. This study aims to assess participants’ sympathy and anger in response to the presentation of depressive symptoms, their perceptions of the controllability of these symptoms, and moreover to examine whether ‘trait’ empathy predicts differences in adolescents’ DL, and likelihood of having engaged in help-giving responses.

To further the investigation of adolescents’ DL and help-giving behaviour, it also may be useful to explore any potential gender differences. Previous Irish and international studies have documented differences between male and female adolescents’ knowledge of mental health and mental illness, whereby females were found to have higher levels of MHL [34, 37, 46, 47] and to be more likely to perceive depression as a serious condition [8] than males, while male adolescents have been found to be less accepting of peers with mental health disorders [42, 48] and to have higher levels of stigma [45, 49] than females. Gender differences have also been documented in adolescents’ help-giving intentions, with previous studies reporting that girls were more likely than boys to suggest intended or hypothetical helping responses that seek the involvement of an adult [12, 37], a professional [50], or ‘another person’ [14]. Building on this literature, the current study aims to further explore gender differences in the likelihood of labelling a cluster of symptoms ‘depression’, reporting having known someone with similar symptoms, and reporting having helped this person.

An exploration of whether adolescents’ DL and help-giving responses differ based on age may also be of value. Age is fundamentally important in all studies that relate to adolescents, because of the vast range of developmental tasks associated with this life stage, and the resulting differences apparent between young people at different points of the adolescent journey [51]. Research has generally demonstrated increases in children and young people’s ability to recognise and understand mental illness with age [52–57]. One reason why older adolescents may have higher DL is that the increasing emergence and prevalence of mental health difficulties, including depression, across the adolescent period [58–61] may result in older adolescents being more likely to interact with and be exposed to same-age peers experiencing mental illness. Adolescents’ intentions to provide help to people experiencing mental illness have also been found to increase among older youth, and older adolescents’ help-giving intentions generally reflect more advanced understanding of appropriate supports [13, 56, 62–64]. The current study aims to further elucidate any age differences in adolescents’ DL, and to explore whether age predicts the likelihood of having known an individual presenting with depressive symptoms and having helped this person.

In sum, the present study therefore builds on the current literature and addresses its existing gaps by examining adolescents’ DL. In addition, the study aims to investigate if adolescents’ DL varies according to their gender, their age, and their empathy; as far as the authors are aware, this study is the first to explore the link between empathy and DL in adolescents. Finally, the study also aims to explore whether adolescents report having known an individual showing symptoms of depression, whether they provided help to this person, and if so, the nature of the help provided. This was achieved through exploring participants’ responses to the presentation of a vignette stimuli describing an adolescent experiencing emotional and behavioural difficulties indicative of depression. The research questions were:

1. Which symptoms do adolescents identify as indicative of the vignette character experiencing difficulties and how do adolescents label the cluster of symptoms displayed by the vignette character?
2. How concerned are adolescents for the vignette character, how much anger and sympathy (state empathy) do adolescents feel towards the vignette character, and how controllable do adolescents believe the character’s symptoms are?
3. How long do adolescents perceive it will take for the character to feel better, and how helpful or harmful do adolescents believe certain responses to the vignette character would be?
4. Do gender, age, and trait empathy predict whether adolescents label a cluster of symptoms ‘depression’?
5. Do adolescents report having known someone with similar symptoms in the past year, and if so, do they report having provided help to this person?
6. Do gender, age and empathy predict whether adolescents report having known someone showing signs of depression, and whether help was provided?
7. What types of help-giving responses do adolescents report having provided to an individual showing signs of depression?

Based on prior research, it is hypothesised that female adolescents, older adolescents, and adolescents with higher trait empathy will be more likely to label the symptoms ‘depression’.

## Methods

### Participants

Participants (*n* = 535) were adolescents residing in Leinster (southeastern province) in Ireland, of whom 277 (51.8%) identified as male and 256 (47.9%) as female. Two (0.4%) participants selected ‘Other’ to describe their gender, and were provided the opportunity to self-describe, with one of these participants self-reporting they identified as “agender” and the other “non-binary”. Participants ranged in age from 12 to 18 years (*M* = 14.90 years, *SD* = 1.58); three (0.5%) did not give their date of birth. Participants’ primary caregivers were asked to give some information on their background when giving consent for their child’s participation in the study. The majority (74.6%) of caregivers of adolescent participants stated that they were born in Ireland and identified as White Irish (77.6%). Almost half (49.9%) reported that the adolescents’ primary female caregiver had completed some level of post-secondary education. For a full breakdown of participants’ demographic characteristics, please see Supplementary File 1.

### Materials

#### Vignette Stimulus

The vignette stimulus can be found in Supplementary File 2. This vignette was adapted from a vignette originally developed and validated by O’Driscoll [65], see O’Driscoll, Heary, Hennessy and McKeague [66–68], and further validated by Silke [69]. An introductory sentence was provided to participants stating that the person described in the vignette is “the same age as you”. The vignette then describes a young person’s behaviour, with six of the nine DSM-IV [70] criteria for depression depicted. However, no diagnostic labels accompanied the vignette. To facilitate the vignette character being sex-matched to participants, two versions of the vignette were provided, with the same character and behaviour described, but named either Michael or Michelle.

#### Mental Health Literacy

An adapted version of the Friend in Need questionnaire [36] was selected for use in the current study, and encompasses assessment of: level of concern, labelling of problem, recognition of symptoms, assessment of prognosis (length of time until recovery), and need for help. This has been used in other studies of adolescent MHL [37, 42, 71, 72]. Beliefs about the helpfulness or harmfulness of various potential responses were assessed by asking participants to mark whether they believe each “person or treatment” would be ‘helpful’, ‘harmful’ or ‘make no difference’ to the vignette character¸ as used in multiple previous studies [30, 50, 56, 73, 74]. Example items are ‘*Make an appointment for Michael to see a GP’* and ‘*Keep Michael busy to keep his mind off his problems’*. These questions do not form a scale; instead, participants’ responses were analysed at an item level.

#### Empathy

‘Trait’ empathy was measured using the 20-item Basic Empathy Scale, which was developed in and has been validated for adolescent samples [75]. Items are rated on a five-point scale from ‘Strongly disagree’ to ‘Strongly agree’. A sample item is “*I get caught up in other people’s feelings easily*”. Four items are worded negatively and are thus reverse-scored. Cronbach’s *α* was 0.827. Participants’ mean trait empathy was 3.70 (*SD* = 0.44; possible range 1–5).

The anger, sympathy, and controllability subscales of the Indicator Questions for Perceived Controllability scale [76] were also used as part of the assessment of state empathy. These subscales measure participants’ anger and sympathy towards the vignette character as well as perceptions of the controllability of the vignette characters’ symptoms. Each subscale comprises three items answered on a nine-point scale. Cronbach’s *α* was 0.616 for the controllability subscale, 0.831 for the anger subscale and 0.751 for the sympathy subscale.

#### Previous Experience of Help-Giving

Participants’ previous experience providing help to someone experiencing depression was assessed by a series of questions used by Yap and Jorm [13]. First, participants were asked, “*In the past year, has anyone in your family or close circle of friends had a problem similar to Michael/Michelle’s?*”, and responded by ticking ‘Yes’, ‘No’, or ‘Don’t Know’. Participants who answered ‘Yes’ were asked, “*Did just one person have the problem, or more than one?*”, and answered by selecting ‘Just one’ or ‘More than one’. Participants who selected ‘More than one’ were directed to a call-out box that contained the following instruction: “*Because you know more than one person who had a problem similar to Michael/Michelle’s, for the next few questions, I want you to think about the person you know best*.” Participants were next asked, “*How old was that person at the time?*” and answered by selecting one of the following options: ‘0–9 years’, ‘10–19 years’, ‘20–29 years’, ‘30–39 years’, ‘40–49 years’, ‘50–59 years’, ‘60 years or over’, ‘Don’t Know’. The next question was “*What was this person’s gender?*”; participants selected ‘Male’, ‘Female’ or ‘Other’. Following this, participants were asked “*Was this person a family member or a friend?*” and selected ‘Family member’ or ‘Friend’. Previous help-giving behaviour was then assessed by asking “*In the past year, have you done anything to help this person?*”. Participants selected ‘Yes’ or ‘No’, after which they were finally asked “*What did you do?*”, which was open-ended and allowed participants to write their response.

### Procedure

Ethical approval for the study was sought and granted from the Trinity College Dublin School of Psychology Research Ethics Committee. Participants were recruited through their schools. Principals of 200 secondary schools across the Leinster region of Ireland were informed about the study by post, of which fourteen schools agreed to facilitate the study. A total of 1,561 study packs were distributed across the 14 schools, which included an information sheet and consent form for the adolescents’ parent/guardian, and an information sheet and assent form for the adolescents themselves. The sample of 535 adolescents therefore represents a 34% response rate. Written informed consent was provided by parents/guardians of all participating adolescents, and adolescents provided written informed assent themselves. Participants selected a sex-matched questionnaire, and completed this questionnaire in hard copy, in a classroom on the premises of each participating school, during school hours and supervised by a member of the research team. Prior to completion of the questionnaires, the researcher reminded all participants to answer questions as honestly as possible, that there were no ‘right’ or ‘wrong’ answers, and that they could withdraw their participation at any time. Participants completed the questionnaire at their own pace and were each provided with a debriefing sheet upon finishing.

### Analysis

Little’s Missing Completely At Random (MCAR) test was found to be significant, *χ*^2^ (9999) = 10,459.394, *p* < 0.001. As there were no variables with 5% or more missing values, the missing data were imputed using the Expectation Maximisation procedure for multiple imputation.

As gender was being used as a grouping variable in analyses, it was decided that the two participants who did not identify as male or female would be excluded from analyses on the basis that the group was too small (less than 1% of the sample), thus leaving a final sample of 533 participants (52% male, 48% female) for analysis purposes.

Responses to open-ended questions (i.e., “*In five words or less, what do you think is the matter with X?*”, “*Please quote all the words/phrases from X’s story that suggest to you that he/she might be experiencing difficulties*”, and ‘*What did you do [to help this person]?*’) were analysed using conventional content analysis, according to the guidelines set out by Hsieh and Shannon [77], using the NVivo programme, version 12 [78].

Quantitative data were analysed using IBM Statistical Package for Social Sciences (SPSS) version 29.0 [79]. A binomial logistic regression was performed to ascertain the effects of participant gender, age, and empathy on the binary outcome variable of labelling the vignette character’s symptoms as depression (0 = did not label as depression; 1 = labelled as depression), while a multinomial logistic regression explored whether participant gender, age, and empathy predicted the likelihood to having known someone showing signs of depression in the last year (response options: yes, no, don’t know). A further binomial logistic regression investigated whether gender, age and empathy predicted whether the participant reported having helped the person (0 = did not help; 1 = helped). A critical significance level of *p* = 0.01 is reported throughout, due to the large number of tests conducted and the subsequent likelihood of Type 1 error.

## Results

### Recognition and Conceptualisation of Depressive Symptoms

Six of the nine DSM-IV (American Psychiatric Association, 2000) symptoms were mentioned in the vignette; participants were asked “*Please quote all the words/phrases from Michael/Michelle’s story that suggest to you that he/she might be experiencing difficulties*” and responses were coded for the presence of words relating to these symptoms. Responses were coded for each symptom mentioned; the most frequently mentioned symptom was depressed mood (68.0%), followed by diminished interest (50.1%), insomnia (35.7%), diminished concentration (20.0%), feelings of worthlessness (18.3%) and fatigue (17.6%). The mean total number of symptoms identified was relatively low, at 2.10 (*SD* = 1.21) out of a possible 6.

Participants were also asked to explain in five words or less what they “*think is the matter*” with the vignette character showing signs of these symptoms. Responses were coded using content analysis; where participants gave multiple explanations, responses were coded for each (e.g., the response “Getting bullied or depressed” was coded for both “bullying” and “depression”). The eight most frequently mentioned explanations, and the percentage of participants who mentioned each, are presented in Table 1. The most frequently mentioned explanation was “depression” or “depressed”, mentioned by 46.7% of participants; note this was only coded when participants specifically used the words “depression” or “depressed”.

**Table 1:** Percentage of participants who mentioned each explanation when asked what they “thought was the matter” with the vignette character

Attributed explanation	Sample quote	Frequency (%)
“Depression”/ “depressed”	“I think Michelle is depressed” (Female, age 12)	46.7
“Problems at home”/problems with family	“Something is wrong at home” (Female, age 14)	12.6
Bullying	“Maybe he is being bullied” (Male, age 15)	7.7
Sad, unhappy, feeling down	“He is feeling a bit down” (Male, age 13)	6.9
Stress, pressure, overwhelmed	“Stressed, overwhelmed” (Female, age 18)	6.8
“Anxiety”/ “anxious”	“I think he is anxious” (Male, age 16)	5.4
Sleep issues, fatigue	“He needs to get sleep” (Male, age 15)	5.1
“Worried”	“She is worried about something” (Female, age 15)	3.8

A binomial logistic regression was performed to ascertain whether participant gender, age, and trait empathy were associated with the likelihood that participants labelled the symptoms as ‘depression’. The Box-Tidwell (1962) procedure found that continuous independent variables were linearly related to the logit of the dependent variable. The logistic regression model was statistically significant, χ^2^(3) = 17.191, *p* < 0.001, however, the model explained just 4.3% (Nagelkerke *R*^*2*^) of the variance. Age (*p* = 0.005) and empathy (*p* = 0.005) were statistically significant predictors of using the label ‘depression’; for every unit increase in age, there was a 1.172 increased in the odds of labelling the symptoms ‘depression’, and for every unit increase in BES score, the odds of identifying depression increased by 1.838, see Table 2.

**Table 2:** Logistic regression predicting likelihood of labelling symptoms ‘depression’ based on gender, age, and empathy

							95% C.I. for Odds Ratio	
	*B*	SE	Wald	*df*	*p*	Odds Ratio	Lower	Upper
Gender^a^	− 0.086	0.190	0.203	1	0.652	0.918	0.633	1.332
Age	0.159	0.057	7.760	1	0.005*	1.172	1.048	1.311
Empathy	0.609	0.218	7.807	1	0.005*	1.838	1.199	2.816

Reference category for dependent variable = did not label as depression

^a^Reference category = male; *p < 0.01

### Level of Concern, Sympathy, and Anger, and Perceptions of Symptom Controllability

Participants showed high levels of concern (*Mdn* = 3.00, possible range 1 [“not at all worried”] – 4 [“extremely worried”]), high levels of sympathy (*M* = 7.52, *SD* = 1.25, possible range 1–9) and low levels of anger (*M* = 2.35, *SD* = 1.51, possible range 1–9) towards the vignette character. Participants perceived the vignette character’s problems to be relatively outside the character’s control (*M* = 3.93, *SD* = 1.55, possible range 1–9, with 9 indicating the problems are completely under the character’s personal control).

### Beliefs About Prognosis, and Beliefs About the Helpfulness of Actions

Most participants believed it would take at least 1–2 months for the character to feel better (*Mdn* = 3.00, possible range 1 [“1–2 days”] – 4 [“Longer than a few months”]). The vast majority (92.1%) believed the vignette character needed the help of another person. When asked about certain help-giving actions a person could take, almost all (97.7%) participants believed that “*listening to Michael/Michelle’s problems in an understanding way*” would be helpful. However, more variation in beliefs was reported for certain actions: while just over half (54.6%) of adolescents thought that “*making an appointment for Michael/Michelle to see a GP*” would be helpful, 28.0% believed that this would be a harmful response. The majority (52.7%) of adolescents believed that asking the vignette character if they were feeling suicidal would be harmful, while 34.8% thought this would be helpful, and 12.5% thought this would make no difference. The percentage of participants who believed each action as ‘helpful’, ‘harmful’, or would ‘make no difference’ is depicted in Table 3.

**Table 3:** Proportion of participants that thought each action would be ‘helpful’, ‘harmful’, or would ‘make no difference’

Response	Helpful (%)	Harmful (%)	Make no difference (%)
Listen to X^a^’s problems in an understanding way	97.7	0.4	1.9
Talk to X firmly about getting his/her act together	16.3	72.0	11.8
Suggest X seek professional help	69.3	13.6	17.0
Make an appointment for X to see a GP	54.6	28.0	17.4
Ask X whether he/she is feeling suicidal	34.8	52.7	12.5
Suggest X has a few drinks to forget his/her troubles	6.9	83.6	9.5
Rally friends to cheer X up	75.0	10.3	14.8
Ignore X until he/she gets over it	1.1	91.4	7.5
Keep X busy to keep his/her mind off his/her problems	69.0	7.1	23.9
Encourage X to become more physically active	76.3	3.9	19.8

^a^X refers to Michael or Michelle

### Reported Exposure to a Family Member or Friend Showing Similar Signs of Depression in the Past Year

After reading the vignette, participants were asked “*In the past year, has anyone in your family or close circle of friends had a problem similar to Michael/Michelle’s?*”. Almost half (49.7%) of participants selected ‘*No*’ as their response, with 38.2% selecting ‘*Yes*’ and 12.0% choosing ‘*Don’t Know’*. Ten participants did not respond to this question. A multinomial logistic regression was conducted to assess whether age, gender, and trait empathy were associated with the likelihood that participants reported having known someone showing signs of depression, which was statistically significant, χ^2^(6) = 43.489, *p* < 0.001, Nagelkerke *r*^2^ = 0.093, see Table 4 for results pertaining to participants who answered ‘Yes’ (reference category ‘No’) Age and empathy were statistically significant predictors of answering “Yes” to this question; each year’s increase in age was associated with 1.231 greater odds of having known someone showing similar symptoms to the vignette character, and each unit increase in empathy was associated with 1.907 greater odds of the same.

**Table 4:** Multinomial logistic regression predicting likelihood of having known someone showing signs of depression in the past year

								95% C.I. for Odds Ratio	
		*B*	SE	Wald	*df*	*p*	Odds Ratio	Lower	Upper
Yes	Gender = Male	− 0.327	0.205	2.547	1	0.111	0.721	0.482	1.078
	Age	0.208	0.063	10.923	1	< 0.001*	1.231	1.088	1.393
	Empathy	0.645	0.239	7.285	1	0.007*	1.907	1.193	3.047

Reference category for dependent variable = “No” (did not know someone who showed signs of depression in past year)

*p < 0.01

Remaining analyses were conducted solely with participants who selected ‘*Yes*’, indicating they had been exposed to an individual showing signs of depression. These participants were asked “*Did just one person have the problem, or more than one?*”; the majority (59.8%) stated ‘*Just one*’, with 40.2% stating, ‘*More than one*’. Those who selected ‘*More than one*’ were told ‘*Because you know more than one person who had a problem similar to Michael/Michelle’s, for the next few questions, I want you to think about the person you know best*’.

### Characteristics of the Family Member or Friend Showing Signs of Depression

Participants were next asked the individual’s age and gender, and whether the individual was a family member or friend. The majority indicated that the person was aged between 10–19 years (78.0%), with 1.5% reporting that the person was younger than 10, 18.5% reporting the person was aged 20–59 years, 1.5% reporting the person was aged 60 + years, and 0.5% reporting they did not know the person’s age. The majority of participants also indicated the person was female (62.6%; with 36.4% reporting the person was male and 1.0% reporting the person did not identify as male or female), and a friend (54.8%).

### Participants’ Help-Giving Responses

When asked, ‘*In the past year, have you done anything to help this person?*’, the majority (88.4%) of participants answered, ‘*Yes*’. Participants that answered ‘*Yes*’ will henceforth be referred to as ‘helpers’, for simplicity. A binomial logistic regression was performed to ascertain whether participant gender, age, and empathy were associated with the likelihood that participants indicated they had helped the person. The logistic regression model was statistically significant, χ^2^(3) = 21.227, *p* < 0.001, Nagelkerke *R*^*2*^ = 0.199. Empathy (*p* =  < 0.001) was a statistically significant predictor of answering ‘Yes’ to having helped the person; for every unit increase in BES score, the odds of being a helper increased by 11.910, see Table 5.

**Table 5:** Logistic regression predicting likelihood of reporting having helped the person, based on gender, age, and empathy

							95% C.I. for Odds Ratio	
	*B*	SE	Wald	*df*	*p*	Odds Ratio	Lower	Upper
Gender^a^	− 0.640	0.546	1.377	1	0.241	0.527	0.181	1.536
Age	0.110	0.169	0.426	1	0.514	1.117	0.802	1.555
Empathy	2.477	0.608	16.598	1	< 0.001*	11.910	3.617	39.219

Reference category for dependent variable = “No” (did not help the person)

^a^Reference category = male

*p < 0.01

Helpers were next asked ‘*What did you do [to help this person]?*’, and were provided an open-ended response option. Coding of responses identified eleven categories of help-giving behaviours, see Table 6. If a response detailed more than one help-giving behaviour, and these fell into different categories, it was coded for each. Help-giving behaviour that included comforting, reassuring, listening, and talking to the person was the most frequently referenced category, with three-quarters (76.4%) of helpers’ responses mentioning this.

**Table 6:** Categories of help-giving responses referenced by participants

Category	Sample quote	Frequency (%)
Comforted and talked to the person	“I always talked to them, I did my best to comfort them, I reassured her that it will get better and we had long deep talks about the situation” (Male, aged 16)	76.4
Encouraged professional care	“Encouraged them to speak to professional” (Female, aged 18)	10.6
Encouraged self-care	“Tried to encourage him to go to bed earlier so he can get up for school and not be aggravated” (Female, aged 15)	9.5
Informed/engaged an adult	“Informed members of staff who helped her” (Female, aged 13)	9.0
Encouraged positive thinking	“Try motivate her to look at the positive side of life” (Male, aged 15)	8.0
Distracted	“Clear their head by distracting them with things they like” (Male, aged 16)	5.5
Active involvement in professional care	“Spoke to him, went to counselling session with them” (Female, aged 17)	4.5
Discouraged negative actions	“Tried to get her to stop drinking, tried to talk her out of self-harming” (Male, aged 16)	3.0
Dismissed	“Told him to get over it” (Male, age 14)	3.0
Monitored behaviour	“Watched them closely for next few weeks but never directly mentioned it to that person” (Male, aged 16)	2.5
Practical help	“I took care of her children” (Female, aged 15)	2.5
Talked to peers	“Discussed with friends” (Female, aged 16)	1.0

## Discussion

This study aimed to extend the literature on adolescent MHL, by focusing specifically on depression literacy (DL) and exploring how this may vary as a function of adolescents’ gender, age, and level of empathy. It is the first study to explore the link between empathy and DL in adolescents. The study also examines adolescents’ self-reported experiences of helping an individual showing symptoms of depression, one of few studies to report on actual (rather than hypothetical or intended) help provided.

Approximately half (46.7%) of participants in the current study labelled the vignette character as ‘depressed’. This rate of labelling is in line with the results of a systematic review and narrative meta-synthesis, which found that, on average, 40.8% of young people labelled depression across studies [34]. However, this is higher than that found in a previous study with Irish adolescents, in which 32.52% of participants labelled a similar (but not identical) vignette as ‘depressed’ [37]. In the current study, with every year increase in age for adolescents, the odds of labelling the symptoms ‘depression’ increased by 17.2%. Previous research has also noted increases in children and adolescents’ ability to recognise mental illness with age [52–56], possibly due to the emergence of these conditions later in adolescence [58]. Also, with age comes greater social opportunities to engage with peers across a variety of contexts and more intimate friendships [e.g., 80], as well as the potential for greater exposure to mental health promotion activities in school (and elsewhere in the community) over time [81], which may increase MHL.

It could be seen as concerning that over half of participants did not identify depression, and that the mean number of symptoms identified was also relatively low, at 2.10 out of a possible 6 depicted in the vignette. However, as noted earlier, some researchers have cautioned against interpreting research findings based solely on adolescents’ ability to recognise labels aligned with diagnostic criteria typically used by mental health professionals, suggesting this essentially reduces the construct of MHL to “knowledge of the contents of Diagnostic and Statistical Manual of Mental Disorders” [82]. It has been argued that this may lead to a focus on biomedical explanations of mental illness, overlooking the role of psychosocial predictors [21]. As such, it is perhaps more important to note that the vast majority (92.1%) of participants believed the vignette character needed the help of another person. Participants also showed high levels of concern and sympathy, and low levels of anger towards the vignette character, perceiving their problems to be relatively outside their control. Furthermore, approximately 32.2% of adolescents suggested situational factors (such as problems at home, bullying, stress, and sleep issues) were involved when asked “what was the matter” with the vignette character. Together, these results highlight participants’ nuanced emotional responses towards the vignette character, as well as a level of appreciation of the complex nature of mental health issues (including the role of potential external factors), and recognition of the need for support.

Approximately one third (34.8%) of participants thought that asking the vignette character if they were feeling suicidal would be helpful, in line with a previous Australian study where 33% of adolescent males and 38% of adolescent females endorsed the action’s helpfulness [74]. These results stand in contrast with a study of Iranian adolescents, where just 11.1% of boys and 14.0% of girls thought this would be helpful [57], and a Japanese study where just 7% of adolescent participants rated this action as helpful [83]. However, it is important to highlight that over half (52.7%) of participants in the current study believed that asking about suicide would be harmful. Although more research is required regarding the safety of lay assessments of suicide risk, systematic reviews suggest that asking about suicide-related behaviours, including suicidal ideation, in research assessments is not harmful [e.g., 84]. Indeed, a previous review concluded that asking about suicide may reduce, rather than increase, suicidal ideation [85], and suicide prevention experts [86, 87] endorse the helpfulness of this action.

About four-in-ten (38.2%) participants reported that, in the past year, someone in their family or close circle of friends had a problem similar to that depicted in the vignette. These individuals were predominantly female same-aged friends, aligning with the higher prevalence of depression among female adolescents [88]. The results of the current study also confirm that informal help-giving regularly occurs amongst adolescents; of those participants who reported having known someone showing signs of depression, 88.4% stated that they had done something to try and help this person. Comforting, reassuring, listening, and talking to the person was the most frequently referenced category of support provided. Although previous research has characterised these kinds of responses offered by young people as helpful, or indicative of greater MHL, further research is required to more comprehensively understand the nature and impact of adolescents’ help-giving, from the perspectives of the help-giver and help-recipient.

While 69.3% of all participants stated that they believed that suggesting the vignette character seek professional support would be helpful, just 10.6% of participants who reported actual help-giving behaviour indicated that they encouraged professional care. This inconsistency between beliefs and behaviour is also observed in help-seeking behaviour for one’s own mental health issues. For example, a large-scale German study found that most participants recommended seeking help from a professional but a much lower proportion actually engaged in this behaviour [89]. Indeed, the gap between beliefs, intentions, and actual behaviour is well-established in many domains [e.g., 90], but its manifestation in this context warrants further and more comprehensive exploration.

Similarly, just 9.0% of helpers reported having informed an adult. Research with Australian adolescents reports that a significant number of participants stated that they would not personally recruit an adult’s help, or encourage a peer to connect with an adult, whether the peer displayed depression with [11] or without [12, 74] suicidal thoughts. Adolescents frequently cite concerns about broken confidentiality with regards to their mental health [e.g., 91], in particular with regards to information being shared with their parents [34]. This implied contract of confidentiality in adolescent friendships may therefore act as a deterrent to young people informing an adult of concerns that they have regarding a friend’s mental health.

It is important to note that there are several limitations to this study that may impact on the generalisability of findings. The use of a vignette may limit the ecological validity of the study’s assessment of DL, as characters and situations may not have seemed real or relevant to participants [72]. A single vignette represents just one situation and one particular constellation of symptoms, and other situational and relationship factors that cannot be captured in a vignette may have greater predictive power in real-life situations. Furthermore, adolescents’ age, empathy, and gender predicted just 4% of the variance in labelling symptoms as depression, suggesting that this outcome is instead better predicted by a different set of unmeasured determinants. The selection of specific variables for measurement in the current study was based on empirical justification, balanced with resources available and the ethical responsibility to reduce participant burden. Nonetheless, it is vital that further research explores the role of other potential predictors. In addition, the vignette character’s sex was matched to the sex of each adolescent participant (i.e., male adolescents read about Michael and female adolescents read about Michelle), and participants were told that the vignette character was “the same age as you”. Although adolescents’ friendships tend to be governed by homophily in many domains including sex and age [92], an interesting line of future research could investigate whether adolescents’ understanding of depressive symptoms differs based on the sex, age, or other characteristics of the individual displaying these symptoms.

The wording of certain questions may have primed participants towards certain responses. For example, participants were asked “What do you think is *the matter* with Michael/Michelle?” (emphasis added); worded differently, some participants may not have interpreted the vignette as indicative of anything “wrong”. Participants were also asked if they knew someone with “a problem similar to Michael/Michelle’s”; as the vignette depicted an adolescent, this may have influenced participants to only think about adolescents and not individuals of other ages. Other research has explored hypothetical help-giving in response to a vignette, and as such, the fact that the current study reports participants’ actual previous help-giving behaviour is a strength. As this behaviour was self-reported, it may therefore be biased; adolescents may have been more likely to report their behaviour in positive terms. However, research has suggested that socially desirable responding is not associated with adolescents’ self-reported prosocial behaviour [93]. It is also important to note that the help provided was reported from the help-giver’s perspective, meaning it is not possible to ascertain whether the help provided was actually experienced as helpful or appropriate by the recipient. This may be an important direction for future research.

The results of this study have important implications for guiding efforts to improve adolescent DL, as well as the support that adolescents may provide to young people experiencing symptoms of depression. For example, the results suggest that psychoeducational interventions that (a) effectively focus on how to initiate conversations and take appropriate action, targeting the belief-behaviour gap, (b) reinforce the safety of assessing suicide risk, and (c) underscore the importance of involving adults, may be most needed. Empathy also emerged as a key predictor, which is optimistic given that research suggests empathy may be modifiable through training, and that receipt of empathy training is effective in increasing adolescent helping behaviour [e.g., 94], therefore positioning this construct as a suitable target for intervention in this context. Moreover, the findings suggest that younger adolescents may stand to benefit most from such interventions.

### Summary

This study advances knowledge of adolescents’ DL, contributing valuable insights into the recognition and conceptualisation of depressive symptoms. The study is the first to explore how adolescents’ DL may be associated with empathy, and one of the first to report adolescents’ actual help-giving behaviour in this context. The results revealed that older adolescents and those with higher ‘trait’ empathy were more likely to label a cluster of symptoms as ‘depression’, and more likely to report having known someone who displayed similar symptoms in the past year; empathy was a particularly strong predictor. Overall, adolescents showed high levels of concern and sympathy and low levels of anger, with the vast majority believing a person experiencing these symptoms would require help from another person. Amongst those with experience providing help to a person showing signs of depression, the most frequently-reported helping response was comforting and talking. Overall, this study identifies much capability among adolescents, and reinforces the need for tailored and developmentally-sensitive interventions that encourage empathy, in order to harness the potential benefits of increased DL.

## Supplementary Information

Below is the link to the electronic supplementary material.

Supplementary file1 (PDF 122 KB)

Supplementary file2 (PDF 116 KB)

## Data Availability

Consent was not sought from participants to archive their data, so regrettably there is no way to share the data from this study.
