# Supplementary material for: Depression Literacy and Self-Reported Help-Giving Behaviour in Adolescents in Ireland
Source: Child Psychiatry Hum Dev. 2024 Jun 25;57(2):543–55. doi: 10.1007/s10578-024-01727-w (PMC13128722; doi:10.1007/s10578-024-01727-w)
Supplement: Supplementary file 1 — Supplementary file1 (PDF 122 KB) [file 10578_2024_1727_MOESM1_ESM.pdf]

Supplementary File 1

Article title:

Depression literacy and self-reported help-giving behaviour in adolescents in Ireland

Journal name:

Child Psychiatry and Human Development

Author names:

Sadhbh J. Byrne[1,2,*], Lorraine Swords[2], Elizabeth Nixon[2]

Affiliations:

[1]Department of Psychology, Maynooth University, Maynooth, Ireland

[2]School of Psychology, Trinity College Dublin, Dublin, Ireland

E-mail address of corresponding author:

[*]Corresponding author : sadhbh.byrne@mu.ie

Table 1

Descriptive data for adolescents' gender and age, caregivers' place of birth, caregivers' ethnic background, and primary female caregiver's highest level of education completed to date.

**Variable**		**%**
Age – *M* (*SD*)		14.9 (1.58)
Gender	Male	51.8
	Female	47.9
	'Other'	0.4
Where primary caregiver was born	Ireland	74.6
	United Kingdom (UK)	6.0
	Europe (other than Ireland or UK)	3.6
	Asia	1.7
	North America	1.3
	Australia	0.2
	Africa	1.3
	Missing	11.4
Ethnic background[a] of primary caregiver	White Irish	77.6
	Irish Traveller	.2
	Any other White background	5.2
	Black Irish	1.7
	African	.9
	Any other Asian background	1.7
	Other	.4
	Missing	12.3
Primary female caregivers' highest level of education completed to date	Primary or less	2.6
	Junior Certificate	10.8

	Leaving Certificate	23.0
	Diploma/Certificate	29.3
	Primary Degree	10.3
	Postgraduate/Higher Degree	10.3
	Missing	13.6

[a]The categories provided for ethnic background were aligned with those used by the Central Statistics Office in Ireland.
