# Supplementary material for: Depression Literacy and Self-Reported Help-Giving Behaviour in Adolescents in Ireland
Source: Child Psychiatry Hum Dev. 2024 Jun 25;57(2):543–55. doi: 10.1007/s10578-024-01727-w (PMC13128722; doi:10.1007/s10578-024-01727-w)
Supplement: Supplementary file 2 — Supplementary file2 (PDF 116 KB) [file 10578_2024_1727_MOESM2_ESM.pdf]

Supplementary File 2

Article title:

Depression literacy and self-reported help-giving behaviour in adolescents in Ireland

Journal name:

Child Psychiatry and Human Development

Author names:

Sadhbh J. Byrne[1,2,*], Lorraine Swords[2], Elizabeth Nixon[2]

Affiliations:

[1]Department of Psychology, Maynooth University, Maynooth, Ireland

[2]School of Psychology, Trinity College Dublin, Dublin, Ireland

E-mail address of corresponding author:

[*]Corresponding author : sadhbh.byrne@mu.ie

Vignette stimuli

You are now going to read about a teenager called Michael, who is the same age as you. You will be asked to answer some questions about what you think and feel about Michael.

Michael used to enjoy lots of hobbies, such as hanging out with his friends and playing football for his local team. Although Michael sometimes complained about how much time the team had to spend training, he was usually quite good at turning up for practice. A few months ago, Michael started missing a lot of training sessions and has since stopped coming altogether. Over this time, Michael's friends have noticed a change in him, in that he doesn't seem to be interested in doing things with them anymore as he no longer makes an effort to hang out with them or talk to them online. Some of Michael's close friends have also noticed that he seems more irritable lately and doesn't smile, or laugh, or appear to find as much enjoyment in things as he used to. Michael has also started to experience some problems at school. Over the past six weeks or so, Michael has been constantly late for school and has started to fall behind on his school work. When the head teacher asked Michael about this, he said that he has been finding it extremely difficult to get to sleep at night and feels tired all the time. Michael explained that although he would like to do better in school, he just couldn't concentrate on things or think as well as he used to. Michael said that he just feels 'down' all the time now and doesn't think that he is good at anything anymore.

OR

You are now going to read about a teenager called Michelle, who is the same age as you. You will be asked to answer some questions about what you think and feel about Michelle.

Michelle used to enjoy lots of hobbies, such as hanging out with her friends and playing football for her local team. Although Michelle sometimes complained about how much time the team had to spend training, she was usually quite good at turning up for practice. A few months ago, Michelle started missing a lot of training sessions and has since stopped coming altogether. Over this time, Michelle's friends have noticed a change in her, in that she doesn't seem to be interested in doing things with them anymore as she no longer makes an effort to hang out with them or talk to them

online. Some of Michelles's close friends have also noticed that she seems more irritable lately and doesn't smile, or laugh, or appear to find as much enjoyment in things as she used to. Michelle has also started to experience some problems at school. Over the past six weeks or so, Michelle has been constantly late for school and has started to fall behind on her school work. When the head teacher asked Michelle about this, she said that she has been finding it extremely difficult to get to sleep at night and feels tired all the time. Michelle explained that although she would like to do better in school, she just couldn't concentrate on things or think as well as she used to. Michelle said that she just feels 'down' all the time now and doesn't think that she is good at anything anymore.
